# Predicting Unfavorable Pregnancy Outcomes in Polycystic Ovary Syndrome (PCOS) Patients Using Machine Learning Algorithms

**DOI:** 10.3390/medicina60081298

**Published:** 2024-08-11

**Authors:** Raluca Mogos, Liliana Gheorghe, Alexandru Carauleanu, Ingrid-Andrada Vasilache, Iulian-Valentin Munteanu, Simona Mogos, Iustina Solomon-Condriuc, Luiza-Maria Baean, Demetra Socolov, Ana-Maria Adam, Cristina Preda

**Affiliations:** 1 Department of Mother and Child Care, “Grigore T. Popa” University of Medicine and Pharmacy, 700115 Iasi, Romania; raluca-anamaria-i-mogos@d.umfiasi.ro (R.M.); tanasaingrid@yahoo.com (I.-A.V.);; 2 Surgical Department, Faculty of Medicine, “Grigore T. Popa” University of Medicine and Pharmacy, 700115 Iasi, Romania; 3 Clinical and Surgical Department, Faculty of Medicine and Pharmacy, ‘Dunarea de Jos’ University, 800216 Galati, Romania; 4 Endocrinology Department, “Grigore T. Popa” University of Medicine and Pharmacy, 700115 Iasi, Romania; simona.mogos@umfiasi.ro (S.M.);

**Keywords:** PCOS, obstetrical complications, machine learning, prediction

## Abstract

*Background and Objectives*: Polycystic ovary syndrome (PCOS) is a complex disorder that can negatively impact the obstetrical outcomes. The aim of this study was to determine the predictive performance of four machine learning (ML)-based algorithms for the prediction of adverse pregnancy outcomes in pregnant patients diagnosed with PCOS. *Materials and Methods*: A total of 174 patients equally divided into 2 groups depending on the PCOS diagnosis were included in this prospective study. We used the Mantel–Haenszel test to evaluate the risk of adverse pregnancy outcomes for the PCOS patients and reported the results as a crude and adjusted odds ratio (OR) with a 95% confidence interval (CI). A generalized linear model was used to identify the predictors of adverse pregnancy outcomes in PCOS patients, quantifying their impact as risk ratios (RR) with 95% CIs. Significant predictors were included in four machine learning-based algorithms and a sensitivity analysis was employed to quantify their performance. *Results*: Our crude estimates suggested that PCOS patients had a higher risk of developing gestational diabetes and had a higher chance of giving birth prematurely or through cesarean section in comparison to patients without PCOS. When adjusting for confounders, only the odds of delivery via cesarean section remained significantly higher for PCOS patients. Obesity was outlined as a significant predictor for gestational diabetes and fetal macrosomia, while a personal history of diabetes demonstrated a significant impact on the occurrence of all evaluated outcomes. Random forest (RF) performed the best when used to predict the occurrence of gestational diabetes (area under the curve, AUC value: 0.782), fetal macrosomia (AUC value: 0.897), and preterm birth (AUC value: 0.901) in PCOS patients. *Conclusions*: Complex ML algorithms could be used to predict adverse obstetrical outcomes in PCOS patients, but larger datasets should be analyzed for their validation.

## 1. Introduction

Polycystic ovary syndrome (PCOS) is a complex disorder that is defined by excessive levels of androgens, ovarian dysfunction, and the presence of polycystic ovarian morphology on ultrasound examination [1]. PCOS is not just a reproductive disorder but also a syndrome with metabolic repercussions that can impact women’s health at various periods of their reproductive and post-reproductive lives [2]. This impact translates into the occurrence of infertility, cardiovascular diseases, endometrial dysfunction, insulin resistance, and obesity [3].

Numerous studies have been conducted on the maternal, neonatal, and obstetric complications associated with women diagnosed with PCOS, and many of them were included in three meta-analyses that indicated an increased risk of pregnancy complications for this type of patient [4,5,6]. Qin et al. conducted a meta-analysis that analyzed data from a total of 27 trials, which consisted of 4982 women diagnosed with PCOS and 119,692 controls, in order to establish the risk of pregnancy and neonatal complications [6]. The authors concluded that PCOS patients had a significantly increased risk of developing gestational diabetes mellitus (GDM), pregnancy-induced hypertension (PIH), preeclampsia, and preterm birth compared to those without PCOS [6]. Moreover, they demonstrated that newborns had a lower birth weight and a greater likelihood of being admitted to a neonatal intensive care unit when compared to the control group.

The long-term effects of PCOS on women’s health have been the subject of a systematic review conducted by Cooney et al. [7]. It indicated that a diagnosis of PCOS increased the risk of dyslipidemia and cardiovascular disorders, stroke, diabetes, or gynecological cancers in women after the age of 40. 

Despite the wealth of published evidence, only a single consensus document has tackled the impact of PCOS on pregnancy. This document concluded that women with PCOS may have an increased risk of adverse outcomes for both themselves and their newborns [8]. The study emphasized that obesity and/or insulin resistance (IR) can worsen the obstetrical risk. Therefore, it is recommended to closely monitor pregnant women with these conditions [8].

When studying pregnancy complications in women with PCOS, it is crucial to accurately define the specific diagnostic characteristics of PCOS. There are four phenotypes of this disorder, each with its own specific hormonal and metabolic abnormalities that can variably impact obstetrical and neonatal outcomes [8]. The first phenotype (A) is characterized by the association between clinical or biochemical hyperandrogenism (HA), ovulatory dysfunction (OD), and polycystic ovarian morphology (PCOM). The second phenotype (B) comprises a mixture of HA and OD, the third phenotype is diagnosed based on the association of HA and PCOM (C), and the fourth phenotype (D) is determined by the association of OD and PCOM [8,9]. 

The impact of obesity, insulin resistance, and metabolic or hormonal abnormalities, which may differ among different PCOS phenotypes, may have a significant influence on infertility rates [10]. A recent cross-sectional study conducted by Elasam et al. included 368 infertile patients with PCOS and showed that phenotype D was the most prevalent among these patients (51.6%), followed by phenotype B (22.6%), phenotype C (18.2%), and phenotype A (7.6%) [11].

The presence of PCOS increases the risk of multifetal gestations and congenital abnormalities. A retrospective population-based cohort study that evaluated over 9.1 million births included 14.882 women diagnosed with PCOS and evaluated the risk of adverse obstetrical and neonatal outcomes [12]. The authors reported an increased risk of preterm rupture of membranes, preterm birth, delivery via cesarean section, maternal–fetal infections, abruptio placentae, multiple gestations, and congenital anomalies in women diagnosed with PCOS.

Recently, artificial intelligence-based technologies have been intensively studied for the prediction of adverse pregnancy outcomes in various clinical scenarios and have demonstrated good predictive performance [13,14,15,16]. Therefore, in this study, we tested the predictive performance of four machine learning-based algorithms for the prediction of adverse pregnancy outcomes in patients diagnosed with PCOS from a tertiary maternity center in Romania.

## 2. Materials and Methods

This study had an observational prospective design and included patients diagnosed with PCOS by a specialized endocrinologist who carried singletons and delivered at “Cuza Voda” Clinical Hospital of Obstetrics and Gynecology, Iasi, Romania, between 25 November 2022 and 10 March 2024.

PCOS was defined according to the Rotterdam consensus by the presence of two of three of the following criteria: oligo-anovulation, hyperandrogenism, and polycystic ovaries (≥12 follicles measuring 2–9 mm in diameter and/or an ovarian volume >10 mL in at least one ovary) [17].

In this study, we excluded cases involving multifetal pregnancies, underaged patients, inaccurate first-trimester gestational age dating, abortions occurring in the first or second trimester, intrauterine fetal demise, loss of follow-up, incomplete medical records, or the mother’s inability to provide informed consent.

The study received ethical approval from the Institutional Ethics Committees of ‘Grigore T. Popa’ University of Medicine and Pharmacy (Approval No. 132/14.12.2021) and the “Cuza Voda” Clinical Hospital of Obstetrics and Gynecology in Iasi, Romania (Approval No. 15515/24.11.2022).

**Figure 1:**
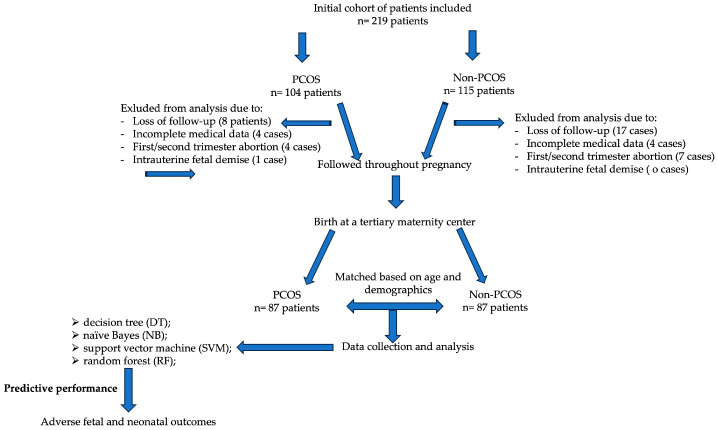
Flowchart that summarizes the study protocol.

The initial study cohort included 219 pregnant patients with (104 patients) or without (115 patients) PCOS (Figure 1). Only the data from 87 patients with PCOS were included in the analysis, and an equal number of selected patients without PCOS constituted the control group using matching characteristics such as age and demographics. 

Comprehensive data were collected from all patients, including demographic information, BMI, smoking status, personal and family history of adverse pregnancy outcomes (such as PE, IUGR, preterm birth, and abruptio placentae), maternal comorbidities, type of delivery, gestational age at birth, birthweight, Apgar scores, and adverse neonatal outcomes (such as NICU admission, intraventricular hemorrhage, necrotizing enterocolitis, and the need for invasive ventilation).

The diagnosis of maternal diabetes mellitus was made by a specialist in diabetes and nutrition based on the American Diabetes Association (ADA) criteria: glycated hemoglobin (A1C ≥ 6.5%) OR fasting plasma glucose (FPG) ≥ 126 mg/dL (7 mmol/L) OR a 2 h plasma glucose ≥ 200 mg/dL (11.1 mmol/L) during an oral glucose tolerance test (OGTT) OR a random plasma glucose ≥ 200 mg/dL (11.1 mmol/L) in patients experiencing classic symptoms of hyperglycemia or hyperglycemic crisis [18]. 

The diagnosis of gestational diabetes mellitus was based on the International Association of the Diabetes and Pregnancy Study Groups (IADPSG) thresholds using a two-hour 75 g OGTT between 24 and 28 weeks of gestation: FPG ≥ 92 mg/dL (5.1 mmol/L) OR one hour plasma glucose ≥ 180 mg/dL (10 mmol/L) OR two-hour plasma glucose ≥ 153 mg/dL (8.5 mmol/mol) [19].

Pregnant individuals are frequently classified as obese or non-obese based on their pre-pregnancy BMI because there is no accepted definition of obesity that is particular to pregnancy. Thus, the diagnosis of obesity was made based on a BMI ≥ 30 kg/m^2^ according to the World Health Organization (WHO) criteria [20].

In the initial phase of our analysis, we employed descriptive statistics and compared categorical variables using Pearson’s χ^2^ test and continuous variables using the Student’s *t*-test between our groups. A *p*-value of less than 0.05 was considered statistically significant. These analyses were conducted using STATA SE (version 17, 2023, StataCorp LLC, College Station, TX, USA).

Also, we used the Mantel–Haenszel test to evaluate the risk of adverse pregnancy outcomes for the PCOS group in comparison with the control group and reported the results as a crude and adjusted odds ratio with a 95% confidence interval (CI). The magnitude of the confounding was calculated using the formula: (crude OR- adjusted OR)/ adjusted OR.

This analysis was followed by the employment of a generalized linear model (GLM) to identify clinical and paraclinical predictors of adverse pregnancy outcomes in patients with PCOS, quantifying their impact as risk ratios (RR) with 95% confidence intervals.

During that last phase, we constructed four predictive algorithms utilizing machine learning methodologies: decision tree (DT), naïve Bayes (NB), support vector machine (SVM), and random forest (RF). These models included important predictors that were identified in the prior analysis. The dataset was divided into a 70% portion for training and a 30% portion for testing. This was then followed by 5-fold cross-validation to ensure a reliable and thorough evaluation.

DT is one of the simplest approaches for data classification. A decision tree is made up of three nodes: a leaf node, an internal node that represents a feature, and a root node that represents a class [21]. The feature is first filtered based on the information it provides. Subnodes are then created for each node based on the feature value. The sample set is located in the root node. A decision sequence can be found on the route that leads from each leaf node to the root node.

The Bayes theorem serves as the foundation for the NB algorithm [22]. Based on past knowledge of the circumstances surrounding an event, this theorem can be used to describe the probability of that occurring [22]. Although features within a class may be interdependent, this classifier makes the assumption that a given feature within that class is not directly related to any other feature.

RF is a combined supervised learning technique made up of various decision trees that each correspond to a different sub dataset [23,24]. Every tree computes the findings and derives the average of the expected outcomes. Using this method, the results variance can be decreased.

SVM is a common algorithm used to perform regression analysis or classification [23]. Using it, training data may be converted into a high-dimensional feature space, and by dividing a hyperplane that offers the least distance between hyperplane points and the highest margin between classes, one can establish a linear optimal solution [23,25].

We performed a sensitivity analysis to evaluate the prediction accuracy of these models for substantially poor pregnancy outcomes in our population. The design and analysis of these models were carried out using Matlab (version R2023a, The MathWorks, Inc., Natick, MA, USA).

## 3. Results

During the initial phase of our analysis, we comparatively evaluated the clinical characteristics of 174 pregnant patients (Table 1). Our data pointed out that the mean age and standard deviation were relatively similar between the examined groups (*p* = 0.52). Also, the smoking status (*p* = 0.19) and the personal history of hypertensive disorders (*p* = 0.24) or autoimmune disorders (*p* = 0.11) did not significantly differ between patients with PCOS and controls.

On the other hand, we found that patients diagnosed with PCOS had significantly higher rates of conception via in vitro fertilization compared with controls (*p* = 0.01). This aspect was correlated with significantly higher rates of nulliparity in the first evaluated group (*p* = 0.009) compared with those identified in the second group.

The mean value and standard deviation for the BMI in patients with PCOS were 30.11 ± 2.92 kg/m^2^, while in the control group they were 23.17 ± 2.78 kg/m^2^ (*p* = 0.04). We also found significantly higher rates of diabetes (*p* = 0.01) and adverse pregnancy outcomes in their obstetrical history (*p* = 0.02) for patients included in the first group compared with controls.

During the subsequent stage of our research, we assessed the likelihood of negative pregnancy outcomes for the PCOS group in contrast to the control group, considering also the influence of confounding variables such as conception via IVF, the presence of obesity or diabetes, and the personal history of adverse pregnancy outcomes. The findings are displayed in Table 2.

Our crude estimates suggested that patients with PCOS had a higher risk of developing gestational diabetes (OR: 5.51, 95%CI: 1.30–17.72, *p* = 0.01) and had a higher chance of giving birth prematurely (OR: 6.15, 95%CI: 1.27–58.33, *p* = 0.009) or through cesarean section (OR: 2.53, 95%CI: 1.29–4.99, *p* = 0.003) in comparison with patients who were included in the control group.

However, the risk of developing preeclampsia (*p* = 0.49), gestational hypertension (*p* = 0.29), intrauterine growth restriction (*p* = 0.69) did not significantly differ between groups when using crude estimates. Also, regarding neonatal outcomes, the odds of NICU admission (*p* = 0.08), invasive ventilation (*p* = 0.46), developing necrotizing enterocolitis (*p* = 0.65), and neonatal deaths (*p* = 0.56) were relatively similar between the evaluated groups in terms of statistical significance.

When adjusting for confounders using the Mantel–Haenszel test, only the odds of delivery via cesarean section remained significantly higher for PCOS patients. The magnitude of the confounding in this case varied between 12 and 18%.

We have also evaluated the significant clinical predictors for the development of gestational diabetes, fetal macrosomia, and preterm birth in PCOS patients using a generalized linear model (Table 3). Our analysis revealed that the conception via IVF increased the risk of gestational diabetes (RR: 3.18, 95%CI: 0.98–18.22, *p* = 0.01), fetal macrosomia (RR: 4.63, 95%CI: 1.22–17.39, *p* = 0.03), and preterm birth (RR: 5.24, 95%CI: 1.74–21.73, *p* = 0.02).

Obesity was outlined as a significant predictor for gestational diabetes (RR: 5.31, 95%CI: 1.16–32.88, *p* = 0.02) and fetal macrosomia (RR: 3.46, 95%CI: 1.19–14.83, *p* = 0.04), but not for preterm birth (*p* = 0.32), while a personal history of diabetes demonstrated a significant impact on the occurrence of all evaluated outcomes (*p* > 0.05).

Last but not least, a personal history of adverse pregnancy outcomes was significantly associated with an increased risk of preterm birth (RR: 3.97, 95%CI: 0.14–10.22, *p* = 0.02).

During the last phase of our investigation, we calculated the predictive performance of four ML-based algorithms that included significant predictors determined using the generalized linear model (Table 4).

Our results indicated that RF performed the best when used to predict the occurrence of gestational diabetes (Se—88.12%, Sp—78.16%, and AUC value: 0.782), fetal macrosomia (Se—88.91%, Sp—81.26%, and AUC value: 0.897), and preterm birth (Se—89.77%, Sp—86.23%, and AUC value: 0.901) in PCOS patients.

The SVM algorithm achieved similar performance just for the prediction of preterm birth (Se—77.78%, Sp—87.38%, and AUC value: 0.883), and not for the other evaluated outcomes. For example, the Se of this algorithm for the prediction of gestational diabetes was only 66.67% and was accompanied by a Sp of 61.17% and an AUC value of 0.693. On the other hand, its predictive performance was better for fetal macrosomia (Se—66.67%, Sp—80.58% and AUC value—0.721).

DT and NB achieved only modest results for the prediction of the evaluated outcomes, with AUC values ranging from 0.625 to 0.778. When comparatively analyzed, NB performed better than DT for the prediction of gestational diabetes (Se—55.55% versus 44.44%, Sp—88.46% versus 87.50%), fetal macrosomia (Se—55.55% versus 44.44%, Sp—88.46% versus 80.58%), and preterm birth (Se—66.67% versus 55.56%, Sp—88.46% versus 87.50%).

## 4. Discussion

Polycystic ovarian syndrome is an intricate hormonal condition observed in women that is regarded as a substantial public health concern in women of reproductive age since its prevalence has risen in the last decade [26]. PCOS has detrimental effects on women’s health throughout their lives. It can lead to a range of health problems, including depressive symptoms, anxiety, low health-related quality of life, fatigue, insulin resistance, abdominal obesity, hypertension, and dyslipidemia [27,28,29,30]. Moreover, the presence of PCOS is a notable factor contributing to infertility in women who are in their reproductive years, mostly because it leads to a lack of ovulation [31].

In this study, our objectives included an evaluation of the risk of adverse pregnancy outcomes in PCOS patients, the identification of specific clinical predictors for these outcomes, and the predictive performance assessment of four ML-based models that included significant clinical predictors.

Our results indicated that patients with PCOS had an increased risk of developing gestational diabetes, fetal macrosomia, or preterm birth and had a higher chance of giving birth through a cesarean section. On the contrary, no significant risk of developing preeclampsia, gestational hypertension, intrauterine growth restriction, or neonatal complications was found for patients with PCOS when using crude estimates.

Several studies have been conducted to compare the pregnancy outcomes of women with PCOS with those who did not have this diagnosis. In the extensive Swedish study conducted by Roos et al. in 2011, it was discovered that pregnant women with PCOS had a more than two-fold increased risk of developing GDM (OR: 2.32, 95% CI: 1.88–2.88) [32]. This association remained significant even after accounting for confounding factors.

A prospective controlled clinical study conducted on a group of individuals with polycystic ovary syndrome found that the incidence of gestational diabetes mellitus was approximately three times greater compared to a control group (14.7% versus 5.3%, respectively) [33].

A recent retrospective cohort included 1357 pregnant women with PCOS and 6940 controls and investigated the rates of obstetrical complications in these patients [34]. Its results indicated that patients with a PCOS diagnosis presented significantly higher rates of gestational diabetes mellitus, hypertension, postpartum hemorrhage, preterm birth, and macrosomia. Moreover, the authors indicated that maternal obesity increased the risk of gestational diabetes, while the use of ART did not increase the risk of any type of obstetrical complications. 

Indeed, the patients with PCOS included in our study had a significantly higher BMI compared with controls, and this could potentially influence the obstetrical outcomes as demonstrated by our generalized linear model. Moreover, these patients had significantly higher rates of IVF conception than those included in the control group, and this could be explained by the fact that our tertiary center is dedicated to monitoring and attending high-risk pregnancies such as those obtained via ART. 

The association between type II diabetes, gestational diabetes, and other endocrinopathies has been established by recent literature data [35,36]. Thus, the oral glucose tolerance test or the determination of glycosylated hemoglobin is mandatory during pregnancy in these patients [37]. However, compliance with these screening strategies during pregnancy and the postpartum period is low, especially in developing countries [38,39]. Hence, it is necessary to implement focused screening programs that include early, prevention-oriented testing and tailored risk assessment. 

A multicenter randomized controlled trial (RCT) was conducted to evaluate the effectiveness of metformin in minimizing pregnancy complications in 274 pregnancies of women with polycystic ovary syndrome [40]. The study found that the incidence of gestational diabetes mellitus was 17.6% in the metformin group and 16.9% in the placebo group. A recent systematic review, conducted by Kanda et al., investigated the effects of metformin administration on both obstetrical outcomes and offspring using data gathered from thirteen studies [41]. The results showed that metformin administration significantly reduced the risk of adverse pregnancy outcomes in patients with PCOS who underwent an IVF procedure and that the effect was more pronounced in those with a high BMI. Moreover, they demonstrated that this intervention was significantly associated with a larger fetal head circumference. These results were marked by moderate heterogeneity, and this suggests the need for more comprehensive studies. 

The literature concerning the likelihood of a cesarean section delivery in women with PCOS is subject to controversy. A recent meta-analysis conducted by Yu et al. in 2016 included 40 observational studies that reported data on a total of 17,816 pregnancies with PCOS. The results from this meta-analysis clearly indicated an increased risk of cesarean delivery in PCOS patients (RR: 1.25, 95% CI: 1.15–1.36, *p* < 0.001). 

However, two additional meta-analyses conducted by Kjerulff et al. in 2011 [5] and Qin et al. in 2013 [6] showed no significant impact of a PCOS diagnosis on the risk of having a cesarean section. With regard to these observations, our data pointed to significantly higher cesarean rates for PCOS patients. This could be explained by the fact that in Romania cesarean delivery rates often exceed 50%, as previously reported [13], our data were collected from a tertiary maternity center that often treats high risk pregnancies, and PCOS patients had significantly higher rates of macrosomic fetuses, which is an indication for this type of intervention.

The association between PCOS and preterm delivery was inconsistently reported in the literature, and a recent meta-analysis that included 42 studies and 7041 women with this disorder outlined a variable likelihood of this outcome depending on geographic area, PCOS phenotypes, and quality of the evaluated studies [42]. Moreover, when the authors adjusted their data according to the BMI, they did not confirm this association. However, their meta-regression indicated a significant risk of preterm birth occurrence in patients who developed gestational diabetes, thus pointing out the need for targeted screening and lifestyle interventions [42]. 

A meta-analysis published in 2024 included 33 studies with a combined sample size of 92,810 patients who underwent an ART procedure in order to establish the most up-to-date association between the PCOS diagnosis and maternal as well as fetal complications. The authors reported a significantly greater likelihood of gestational diabetes with an OR of 1.51 and a 95% CI of 1.17–1.94. PCOS was also associated with an increased risk of preterm delivery, with an OR of 1.29 and a 95% CI of 1.21–1.39. After performing the subgroup analysis, the authors reported a persistently higher risk of gestational diabetes and abortion for patients who underwent fresh embryo transfer (ET). On the other hand, the subgroup of patients who underwent frozen ET had an elevated risk of preterm birth (OR: 1.31, 95%CI: 1.21–1.42). The risk of preterm birth was significantly higher in these patients, regardless of the presence or absence of hyperandrogenism. Hence, it is necessary to monitor cervical length during specific screening programs for timely intervention and preterm birth risk reduction.

In our study, we have shown that conception via IVF, obesity, a personal history of diabetes, and adverse pregnancy outcomes represented significant clinical predictors for the development of gestational diabetes, fetal macrosomia, and preterm birth in PCOS patients. We included these predictors in ML-based algorithms and demonstrated good overall predictive performance only for RF. When used to predict the occurrence of gestational diabetes (Se—88.12%, Sp—78.16%, and AUC value: 0.782), fetal macrosomia (Se—88.91%, Sp—81.26%, and AUC value: 0.897), and preterm birth (Se—89.77%, Sp—86.23%, and AUC value: 0.901) in PCOS patients, this algorithm had the best results compared to DT, NB, and SVM.

As far as we know, this would be the first study in the literature that has calculated their predictive performance. Thus, comparative literature data is missing. However, recent studies have outlined the challenges and opportunities that result from the use of AI-based techniques for the detection of PCOS or for its genetic characterization. For example, a recent systematic review conducted by Barrera et al. investigated the results of 31 studies that identified the utility of algorithms based on AI or ML for the diagnosis or classification of PCOS [43]. The authors found out that the most common ML techniques employed were SVM, K-nearest neighbor, and regression models. The diagnostic accuracies of these algorithms ranged between 89 and 100%, which points out the increased detection rates of PCOS.

Zhou et al. used bioinformatics analysis to characterize the differentially expressed genes (DEGs) between PCOS and non-PCOS patients [44]. The authors found a total of 174 DEGs and 10 hub genes that could serve as therapeutic targets in this category of patients. The functions of the detected genes resumed with protein binding, such as actin, calmodulin, and the ribonucleoprotein complex [44].

It is crucial to consider certain limitations when interpreting the outcomes of this study. The limitations of this study encompass the relatively small patient sample size, the inclusion of a limited number of clinical and paraclinical factors, and the presence of imbalanced data sets. Machine learning algorithms have the ability to perform effectively with small datasets, making them potentially helpful for risk classification. On the other hand, the use of predictive models based on machine learning techniques and the careful planning of our study are significant advantages.

Additional research, involving larger groups of individual pregnancies, could explore different methods of screening using machine learning algorithms. This could provide a more comprehensive understanding of their ability to predict outcomes and their usefulness in clinical settings.

Within our patient cohort, the incorporation of machine learning algorithms stands out for its capacity to encompass multiple confounding variables influencing adverse pregnancy outcomes. This allows for a nuanced risk stratification strategy primarily anchored in clinical determinants. Our data outline the high susceptibility to adverse pregnancy outcomes among individuals with comorbidities such as diabetes, obesity, or prior adverse obstetrical outcomes, underscoring the necessity of modulating these factors to prevent further adverse pregnancy outcomes. Using these algorithms could further enable the timely identification of high-risk patients, commencing as early as the preconception period or initial prenatal consultation, thereby indicating a close follow-up during pregnancy.

## 5. Conclusions

Gestational diabetes, fetal macrosomia, and preterm birth are among the adverse pregnancy outcomes that are significantly associated with PCOS patients, and their significant predictors included conception via IVF, obesity, a personal history of diabetes, and adverse pregnancy outcomes.

The best predictive performance for these outcomes was achieved by the RF algorithm, and supplementary efforts should be made in order to expand the number of clinical and paraclinical predictors that will ultimately help us improve the patients’ risk stratification.

## Figures and Tables

**Table 1:** Clinical characteristics of the studied groups.

Clinical Characteristics	PCOS Group (n = 87 Patients)	Control Group (n = 87 Patients)	*p* Value
Age, years (mean ± SD)	29.6 ± 6.19	26.12 ± 5.74	0.52
IVF conception (n/%)	Yes = 18 (20.68%) No = 69 (79.32%)	Yes = 7 (8.04%) No = 80 (91.96%)	0.01
Nulliparous (n/%)	Yes = 49 (56.32%) No = 38 (43.68%)	Yes = 32 (36.78%) No = 55 (63.22%)	0.009
BMI, kg/m^2^, (mean and standard deviation)	30.11 ± 2.92	23.17 ± 2.78	0.04
Smoking (n/%)	Yes = 3 (3.44%) No = 84 (96.56%)	Yes = 7 (8.04%) No = 80 (91.96%)	0.19
Diabetes (n/%)	Yes = 14 (16.09%) No = 73 (83.9%)	Yes = 4 (6.6%) No = 83 (95.4%)	0.01
Previous diagnosis of chronic hypertension (n/%)	Yes = 5 (5.74%) No = 82 (94.25%)	Yes = 2 (2.29%) No = 85 (97.7%)	0.24
Previous diagnosis of autoimmune disorders (n/%)	Yes = 8 (9.19%) No = 79 (90.8%)	Yes = 3 (3.44%) No = 84 (96.56%)	0.11
Previous diagnosis of adverse pregnancy outcomes (n/%)	Yes = 11 (12.64%) No = 76 (87.36%)	Yes = 3 (3.44%) No = 84 (96.56%)	0.02

Legend: PCOS—polycystic ovarian syndrome, PE—preeclampsia, IUGR—intrauterine growth restriction, SD—standard deviation, n—number of patients, IVF—in vitro fertilization, BMI—body mass index.

**Table 2:** Comparison of the odds of adverse pregnancy outcomes between the evaluated groups controlled for confounding variables.

Adverse Pregnancy Outcomes	PCOS vs. Control Group				Magnitude of the Confounding
	Crude Estimates	*p* Value	M-H Combined	*p* Value **	
	OR and 95% CI		aOR and 95% CI		
Gestational diabetes * IVF	5.51 (1.30–17.72)	0.01	3.12 (0.47–20.58)	0.23	0.77
Gestational diabetes * Obesity			5.01 (0.41–59.65)	0.19	0.10
Gestational diabetes * Diabetes			2.5 (0.25–24.37)	0.61	1.20
Gestational diabetes * Previous APO			5 (0.21–117.89)	0.30	0.10
Fetal macrosomia * IVF	3.71 (0.84–9.03)	0.08	1.59 (0.23–10.57)	0.63	1.33
Fetal macrosomia * Obesity			1.4 (0.14–13.56)	0.77	1.65
Fetal macrosomia * Diabetes			1 (0.10–9.22)	0.99	2.71
Fetal macrosomia * APO			0.87 (0.05–12.97)	0.92	3.26
Preterm birth * IVF	6.15 (1.27–58.33)	0.009	3.92.(0.59–26.10	0.15	0.57
Preterm birth * Obesity			11 (0.64–187.16)	0.07	−0.44
Preterm birth * Diabetes			3.66 (0.35–38.02)	0.27	0.68
Preterm birth * APO			5.5(0.23–128.96)	0.27	0.12
PE * IVF	1.51 (0.16–18.55)	0.49	0.5 (0.06–3.90)	0.51	2.02
PE * Obesity			0.33 (0.03–3.51)	0.36	3.58
PE * Diabetes			0.27 (0.02–2.82)	0.27	4.59
PE * APO			0.16 (0.01–2.56)	0.18	8.44
Gestational hypertension * IVF	1.70 (0.31–11.31)	0.29	0.51 (0.08–3.15)	0.47	2.33
Gestational hypertension * Obesity			0.23 (0.01–3.01)	0.26	6.39
Gestational hypertension * Diabetes			0.18 (0.01–2.28)	0.17	8.44
Gestational hypertension * APO			0.71 (0.22–2.25)	0.56	1.39
IUGR * IVF	1.34 (0.22–9.47)	0.69	0.38 (0.05–2.45)	0.31	2.53
IUGR * Obesity			0.16 (0.01–2.15)	0.15	7.38
IUGR * Diabetes			0.13 (0.01–1.69)	0.10	3.47
IUGR * APO			0.5 (0.15–1.66)	0.25	1.68
Cesarean delivery * IVF	2.53 (1.29–4.99)	0.003	2.15 (1.11–4.15)	0.02	0.18
Cesarean delivery * Obesity			2.25 (1.18–4.30)	0.01	0.12
Cesarean delivery * Diabetes			2.16 (1.13–4.13)	0.01	0.17
Cesarean delivery * APO			2.2 (1.15–4.18)	0.01	0.15
NICU admission * IVF	2.37 (0.78–7.97)	0.08	0.43 (0.04–4.56)	0.48	4.51
NICU admission * Obesity			0.54 (0.04–6.16)	0.62	3.39
NICU admission * Diabetes			0.63 (0.24–1.64)	0.35	2.76
NICU admission * APO			0.35 (0.02–5.10)	0.56	5.77
Need for invasive ventilation * IVF	1.70 (0.31–11.31)	0.46	0.48–13.73	0.26	2.54
Need for invasive ventilation * Obesity			0.23 (0.01–3.01)	0.26	6.39
Need for invasive ventilation * Diabetes			0.18 (0.01–2.28)	0.17	8.44
Need for invasive ventilation * APO			1.14 (0.41–3.15)	0.79	0.49
Necrotizing enterocolitis * IVF	1.51 (0.16–18.55)	0.65	0.5 (0.06–3.90)	0.51	2.02
Necrotizing enterocolitis * Obesity			0.33 (0.03–3.51)	0.36	3.58
Necrotizing enterocolitis * Diabetes			0.27 (0.02–2.82)	0.27	4.59
Necrotizing enterocolitis * APO			0.16 (0.01–2.56)	0.18	8.44
Neonatal death * IVF	2.02 (0.10–120.73)	0.56	0.75 (0.05–9.87)	0.82	1.69
Neonatal death * Obesity			0.6 (0.03–9.15)	0.72	2.37
Neonatal death * Diabetes			0.5 (0.03–7.54)	0.62	3.04
Neonatal death * APO			0.4 (0.02–6.84)	0.53	4.05

Legend: PCOS—polycystic ovarian syndrome, aOR—adjusted odds ratio, CI—confidence interval, M-H—Mantel–Haenszel, PE—preeclampsia, IUGR—intrauterine growth restriction, IVF—in vitro fertilization, APO—history of adverse pregnancy outcomes; *—interaction between outcome and confounding variable, **—*p* value corresponding to the Mantel–Haenszel analysis.

**Table 3:** Results from the generalized linear model for evaluating the impact of clinical predictors on the adverse pregnancy outcomes.

Clinical Predictors	Gestational Diabetes			Fetal Macrosomia			Preterm Birth		
	RR	95%CI	*p* Value	RR	95%CI	*p* Value	RR	95%CI	*p* Value
Age > 35 years	0.76	−0.24–8.56	0.42	0.42	0.03–4.98	0.56	2.19	0.48–12.31	0.06
IVF conception	3.18	0.98–18.22	0.01	4.63	1.22–17.39	0.03	5.24	1.74–21.73	0.02
Nulliparity	0.44	−0.12–9.03	0.59	1.04	0.78–7.78	0.32	0.33	−0.71–8.64	0.86
Obesity	5.31	1.16–32.88	0.02	3.46	1.19–14.83	0.04	2.37	−1.16–11.2	0.32
Smoking status	0.56	−0.76–5.52	0.17	−0.11	−2.31–2.89	0.46	1.12	0.04–3.85	0.05
Diabetes	5.28	0.26–14.95	0.001	6.93	1.32–13.79	0.001	3.14	0.64–9.25	0.03
History of adverse pregnancy outcomes	1.24	0.02–4.86	0.78	1.89	0.71–7.98	0.67	3.97	0.14–10.22	0.02

Legend: RR—relative risk, CI—confidence interval, IVF—in vitro fertilization.

**Table 4:** Comparative analysis of four machine learning algorithms in predicting adverse pregnancy outcomes in PCOS patients.

Models	Gestational Diabetes			Fetal Macrosomia			Preterm Birth		
	Sensibility	Specificity	AUC Value	Sensibility	Specificity	AUC Value	Sensibility	Specificity	AUC Value
DT	44.44	87.50	0.694	44.44	80.58	0.625	55.56	87.50	0.713
NB	55.56	88.46	0.731	55.56	88.46	0.754	66.67	88.46	0.778
SVM	66.67	61.17	0.693	66.67	80.58	0.721	77.78	87.38	0.883
RF	88.12	78.16	0.782	88.91	81.26	0.897	89.77	86.23	0.901

Legend: AUC—area under the curve value, DT—decision trees, NB—naïve Bayes, SVM—support vector machine, RF—random forest.

## Data Availability

Data are available from the correspondent author upon a reasonable request.
